# Making It Last: Storage Time and Temperature Have Differential Impacts on Metabolite Profiles of Airway Samples from Cystic Fibrosis Patients

**DOI:** 10.1128/mSystems.00100-17

**Published:** 2017-11-28

**Authors:** Stephen Wandro, Lisa Carmody, Tara Gallagher, John J. LiPuma, Katrine Whiteson

**Affiliations:** a Department of Molecular Biology and Biochemistry, University of California, Irvine, California, USA; b Department of Pediatrics and Communicable Diseases, University of Michigan Medical School, Ann Arbor, Michigan, USA; University of California, San Francisco

**Keywords:** biomarkers, metabolomics, microbial metabolites, microbiome, reproducibility, sample storage

## Abstract

Metabolites of human or microbial origin have the potential to be important biomarkers of the disease state in cystic fibrosis (CF). Clinical sample collection and storage conditions may impact metabolite abundances with clinical relevance. We measured the change in metabolite composition based on untargeted gas chromatography-mass spectrometry (GC-MS) when CF sputum samples were stored at 4°C, −20°C, or −80°C with one or two freeze-thaw cycles. Daily measurements were taken for 1 week and then weekly for 4 weeks (4°C) and 8 weeks (−20°C). The metabolites in samples stored at −20°C maintained abundances similar to those found at−80°C over the course of 8 weeks (average change in Bray-Curtis distance, 0.06 ± 0.04) and were also stable after one or two freeze-thaw cycles. However, the metabolite profiles of samples stored at 4°C shifted after 1 day and continued to change over the course of 4 weeks (average change in Bray-Curtis distance, 0.31 ± 0.12). The abundances of several amino acids and other metabolites increased with time of storage at 4°C but remained constant at −20°C. Storage temperature was a significant factor driving the metabolite composition (permutational multivariate analysis of variance: *r*^2^ = 0.32 to 0.49, *P* < 0.001). CF sputum samples stored at −20°C at the time of sampling maintain a relatively stable untargeted GC-MS profile. Samples should be frozen on the day of collection, as more than 1 day at 4°C impacts the global composition of the metabolites in the sample.

**IMPORTANCE** Metabolomics has great potential for uncovering biomarkers of the disease state in CF and many other contexts. However, sample storage timing and temperature may alter the abundance of clinically relevant metabolites. To assess whether existing samples are stable and to direct future study design, we conducted untargeted GC-MS metabolomic analysis of CF sputum samples after one or two freeze-thaw cycles and storage at 4°C and −20°C for 4 to 8 weeks. Overall, storage at −20°C and freeze-thaw cycles had little impact on metabolite profiles; however, storage at 4°C shifted metabolite abundances significantly. GC-MS profiling will aid in our understanding of the CF lung, but care should be taken in studies using sputum samples to ensure that samples are properly stored.

## Abstract (precis)

Metabolomics has great potential for uncovering biomarkers of the disease state in CF and many other contexts. However, sample storage timing and temperature may alter the abundance of clinically relevant metabolites. To assess whether existing samples are stable and to direct future study design, we conducted untargeted GC-MS metabolomic analysis of CF sputum samples after one or two freeze-thaw cycles and storage at 4°C and −20°C for 4 to 8 weeks. Overall, storage at −20°C and freeze-thaw cycles had little impact on metabolite profiles; however, storage at 4°C shifted metabolite abundances significantly. GC-MS profiling will aid in our understanding of the CF lung, but care should be taken in studies using sputum samples to ensure that samples are properly stored.

## INTRODUCTION

The staggering metabolic complexity of any human biological specimen results from both human and microbial metabolism and could provide clinically relevant biomarkers of disease. Recent studies based on chromatography and mass spectroscopy have estimated that tens or hundreds of thousands of distinct metabolites are in human biological specimens and that a third to half of them are produced or altered by microbes (1, 2). In this study, we assessed the impact of storage temperature and time on the metabolite composition of sputum samples collected from people with cystic fibrosis (CF). CF is a genetic disease caused by a mutation in a cellular ion transporter that results in increased susceptibility of the respiratory tract to bacterial infection (3–5). The lives of persons with CF are punctuated by periods of worsened respiratory symptoms referred to as pulmonary exacerbations. Although the etiology of these events remains unclear, changes in the structure and/or activity of airway microbial communities are believed to play a role (6–12). Decades of study of the microbiology of CF airway infection have relied on the recovery of selected bacterial species in culture of respiratory specimens. More recently, next-generation DNA sequencing and metabolomic analyses have contributed to a broader appreciation of the complexity and dynamics of the microbial ecology of CF airways (6, 7, 13). Culture-independent approaches have the potential to identify changes in microbial community structure and activity associated with pulmonary exacerbations, thereby offering insight that may lead to novel approaches to the prevention or better treatment of these episodes (14–17).

The utility of metabolomics in advancing our understanding of CF exacerbations, however, depends on an appreciation of how variables in sample handling may impact measurements of global metabolic profiles and/or assessment of specific metabolites of interest. Respiratory samples, including expectorated sputum and bronchoalveolar lavage fluid, are most often obtained from patients in clinical settings where immediate freezing and storage are not practical. Samples may be kept at 4°C or −20°C for various periods of time before being stored at −80°C. Samples may also undergo repeated cycles of freezing and thawing prior to analysis. While studies have addressed the impact of storage temperature on the metabolomic analysis of clinical samples such as plasma and urine (18–20), comparable studies assessing sputum are lacking. We previously examined the impact of storage conditions on CF sputum metabolomic profiles based on liquid chromatography-mass spectrometry (LC-MS) and found that profiles were stable in samples stored for at least 4 weeks at −20°C (21). While LC-MS can be used to study low-molecular-weight compounds, it cannot ionize nonpolar compounds and is not practical for analysis of volatile compounds, which are better analyzed by gas chromatography (GC)-MS. With respect to CF, recent breath sampling has shown that levels of the volatile compound 2,3-butanedione, a pH-neutral bacterial fermentation product, were higher in the breath of CF patients than in that of healthy individuals. Another compound from the same metabolic pathway, 2,3-butanediol, was associated with increased virulence of the CF opportunistic pathogen *Pseudomonas aeruginosa* in both culture-based and murine infection models (22–24). These studies highlight the potential of metabolomic analysis for investigation of the microbiology of airway infection in CF. A critical element of such studies, however, is a more complete understanding of the impact of sample storage on measurements of metabolites detected by GC-MS. In the study reported here, we assessed the stability of a wide range of volatile metabolites in sputum samples stored at 4°C and −20°C for various periods of time with one or two freeze-thaw cycles.

## RESULTS

### Bacterial community profiles.

**FIG 1:**
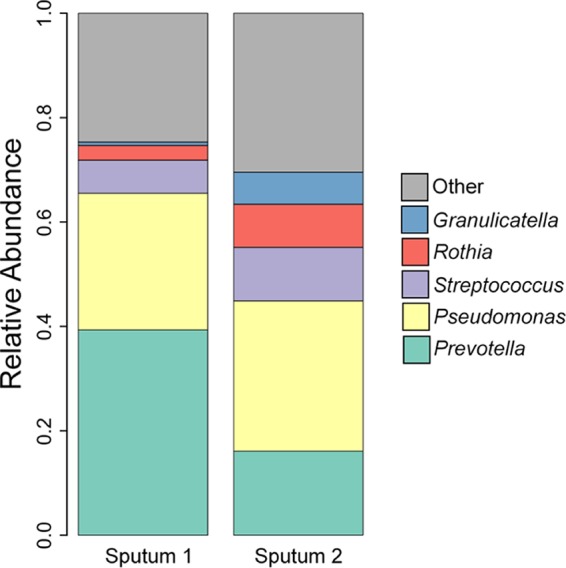
16S rRNA gene sequence profiles of the bacterial communities of the sputum samples aliquoted for the storage study. The V4 region of the 16S rRNA gene was amplified, sequenced, and analyzed with mothur as described previously (21). The relative abundances of the five most abundant taxa in each sputum sample are shown.

Bacterial 16S rRNA gene sequencing revealed similar profiles in two sputum samples collected from two CF patients during the course of routine medical care (Fig. 1). *Prevotella* and *Pseudomonas* dominated in both samples; the distribution of microbes in these samples was typical of an adult CF airway community.

### Effect of storage at 4°C or −20°C.

**FIG 2:**
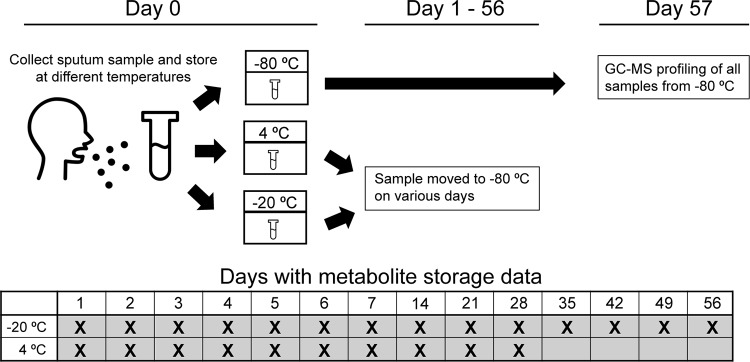
Flowchart and study design schematic. Sputum samples from two patients were homogenized, aliquoted, and stored at either −20°C or 4°C. Duplicates of stored samples were taken daily for a week, and weekly samples were taken for 4 weeks at 4°C and 8 weeks at −20°C. At each time point, an additional replicate was subjected to either one or two freeze-thaw cycles. The aliquots were all analyzed by untargeted GC-MS.

**FIG 3:**
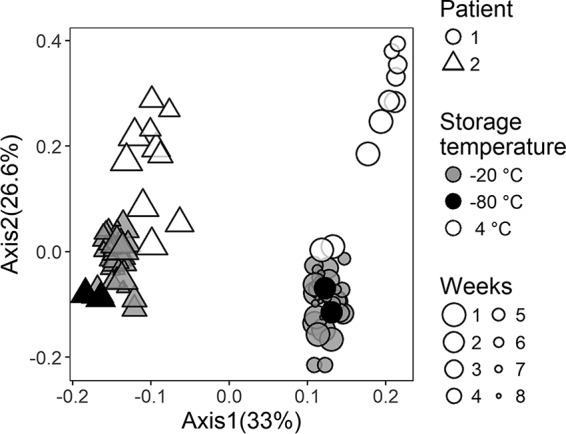
PCoA of BC distances calculated from the metabolite abundances in each sample. The percent variance explained by each axis is shown in parentheses in each axis label. Aliquots of the sample from patient 1 are shown as circles, while those of the sample from patient 2 are depicted as triangles. The gold standard −80°C samples are represented by solid black circles, while −20°C samples are represented by gray circles and 4°C samples are represented by open circles. The size of the symbol reflects the storage time, with later times represented by smaller symbols.

**FIG 4:**
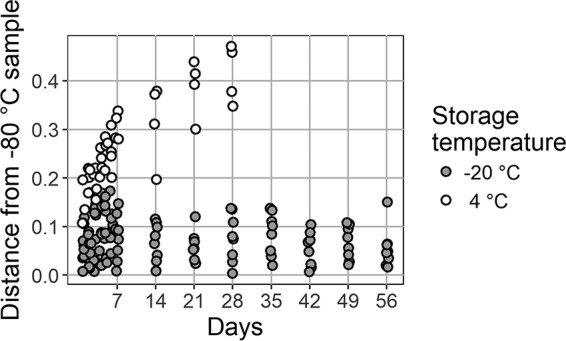
Effect of storage at −20°C versus 4°C. BC distances between samples at each storage temperature and the corresponding patient samples stored immediately at −80°C are shown. All replicates from both samples are shown, including those subjected to one or two freeze-thaw cycles.

Our goal was to determine the impact of storage time and temperature on GC-MS metabolite profiles, as depicted in the flowchart in Fig. 2. Two sputum samples were split into 76 aliquots and stored at 4°C or −20°C, with 2 additional aliquots stored immediately at −80°C; daily measurements were taken for a week and then weekly for 4 weeks (4°C) or 8 weeks (−20°C) (Fig. 2). GC-MS of each sample was performed, and the resulting metabolomic profiles were visualized by principal-coordinate analysis (PCoA) (Fig. 3). A total of 239 metabolites were detected, of which 104 were identifiable. A subset of molecules are represented in a heat map depicting the fold change in metabolite intensity at each storage temperature over time (see Fig. S1 and S2 in the supplemental material). For both patients, the samples stored at −20°C clustered together with the respective time zero samples that were stored immediately at −80°C, while samples stored at 4°C drifted in the ordination space over time. Storage at 4°C versus −20°C was found to significantly affect the overall metabolite profile (permutational multivariate analysis of variance [PERMANOVA]: *r*^2^ = 0.49 [sample 1] and 0.32 [sample 2], *P* < 0.001) (Table S1). Storage at 4°C resulted in larger changes in metabolite abundance over time (Fig. S3). A major contributor to the changing metabolite profile at 4°C was the increasing total ion count between days 1 and 28, which remained stable at −20°C (Fig. S4). The Bray-Curtis (BC) distance of each metabolomic profile from the time zero profile was calculated for samples stored at 4°C or −20°C (Fig. 4). After 1 day, the metabolomic profiles of samples stored at 4°C were observed to be more distant than those of samples stored at 20°C and the average distance from the time zero profile increased each week in samples stored at 4°C. The average distance of the metabolomic profiles of the samples stored at −20°C from the time zero profile remained steady over the course of 8 weeks (average change in BC distance, 0.06 ± 0.04), indicating that the overall metabolite profile did not change with storage at −20°C. In contrast, the metabolite profiles of samples stored at 4°C continued to change over the course of 4 weeks (average change in BC distance, 0.31 ± 0.12).

10.1128/mSystems.00100-17.1 FIG S1  Heat map of a subset of metabolites detected by GC-MS after storage at 4°C. The color scale represents the log_2_ fold difference in metabolite intensity between 4°C and the reference, −80°C. Download FIG S1, PDF file, 0.3 MB. Copyright © 2017 Wandro et al. 2017 Wandro et al. This content is distributed under the terms of the Creative Commons Attribution 4.0 International license.

10.1128/mSystems.00100-17.2 FIG S2  Heat map of a subset of metabolites detected by GC-MS after storage at −20°C. The color scale represents the log_2_ fold difference in metabolite intensity between −20°C and the reference, −80°C. Download FIG S2, PDF file, 0.3 MB. Copyright © 2017 Wandro et al. 2017 Wandro et al. This content is distributed under the terms of the Creative Commons Attribution 4.0 International license.

10.1128/mSystems.00100-17.5 TABLE S1  PERMANOVA of BC distances between samples from each patient based on storage temperature (A) and one versus two freeze-thaw cycles (B). Download TABLE S1, DOCX file, 0.01 MB. Copyright © 2017 Wandro et al. 2017 Wandro et al. This content is distributed under the terms of the Creative Commons Attribution 4.0 International license.

10.1128/mSystems.00100-17.3 FIG S3  Average percent change in each of the 239 metabolites detected over time at 4°C and −20°C. Each point is the average percent change in a single metabolite in both patient samples. Download FIG S3, PDF file, 0.1 MB. Copyright © 2017 Wandro et al. 2017 Wandro et al. This content is distributed under the terms of the Creative Commons Attribution 4.0 International license.

10.1128/mSystems.00100-17.4 FIG S4  Total metabolite ion count (mTIC) of each sample over time colored by storage temperature. Download FIG S4, PDF file, 0.1 MB. Copyright © 2017 Wandro et al. 2017 Wandro et al. This content is distributed under the terms of the Creative Commons Attribution 4.0 International license.

### Metabolites distinguishing patient samples.

**FIG 5:**
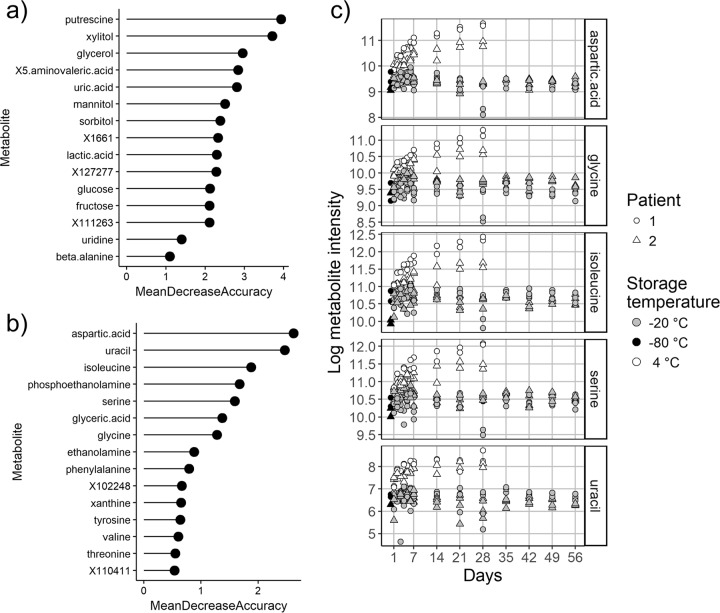
The VIPs from a supervised random-forest analysis to determine which metabolites best distinguish (a) patient 1 and 2 samples and (b) storage at −20°C versus storage at 4°C. All time points were included, and intensities were log transformed. (c) Intensities over time of five metabolites that distinguish storage at the temperatures shown.

The samples collected from each patient clustered separately along the primary PCoA axis (Fig. 3), indicating that the two patients’ sputum samples had unique metabolomics profiles. The metabolites that differed the most between the samples from patients 1 and 2 as determined by supervised random-forest analysis and ranked by the mean decrease in predictive accuracy were putrescine, xylitol, glycerol, 5-aminovaleric acid, and uric acid (Fig. 5A).

### Metabolites that change with storage temperature and time.

The metabolite profiles of sputum samples from both patients stored at 4°C for various lengths of time separate along the secondary PCoA axis, indicating common changes in the overall metabolomic profile with storage at this temperature (Fig. 3). A supervised random-forest analysis was used to determine which metabolites were responsible for the differences between samples stored at 4°C and those stored at −20°C. The top variables of importance (VIPs) separating samples collected at different temperatures are shown in Fig. 5B. Many of the metabolites that were the most different at each storage temperature were amino acids (Fig. 5B and C). The intensities of the VIP metabolites at each time point are shown in Fig. 5C and indicate that the amino acids increased in abundance with time at 4°C and stayed more constant at −20°C.

**FIG 6:**
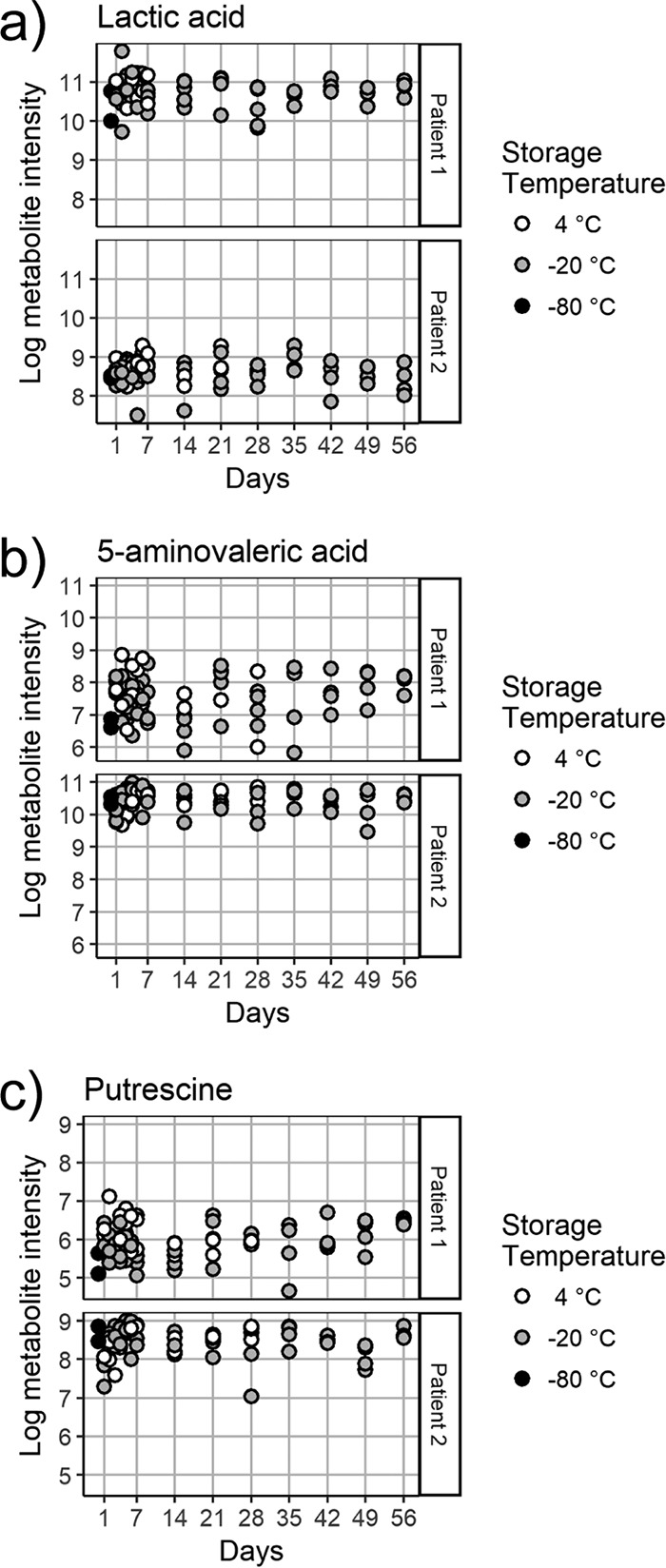
Intensities of several metabolites of potential clinical significance after storage of samples from patients 1 and 2 at 4°C or −20°C, including lactic acid (a), 5-aminovaleric acid (b), and the polyamine putrescine (c).

Clear trends showing either an increase or a decrease over time at either 4°C or −20°C were not observed for several metabolites that have been determined to be clinically relevant to CF in other studies, including lactic acid, putrescine, and 5-aminovaleric acid (Fig. 6).

### Impact of freeze-thawing on metabolomic profiles.

At each time point, samples were assessed after being thawed once and again after a second freeze-thawing. The differences between the overall metabolite profiles of samples thawed once and those thawed twice were not statistically significant (PERMANOVA: *r*^2^ = 0.005, *P* = 0.72; see Table S1).

### Metabolite intensity and CV.

**FIG 7:**
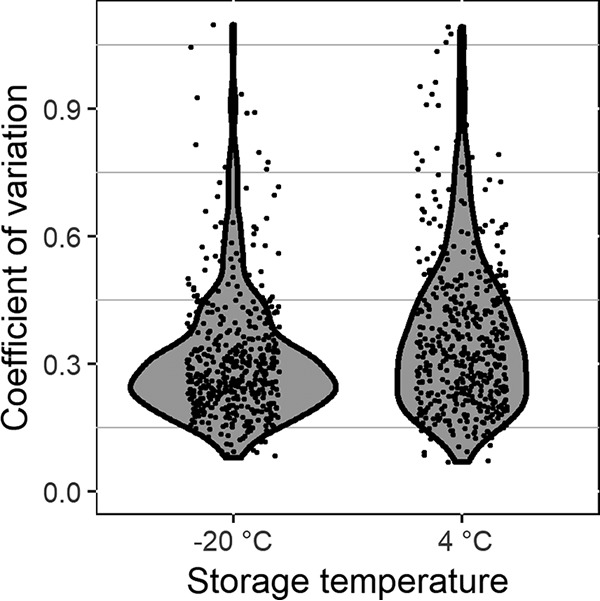
Violin plot of the CVs of each metabolite with storage at 4°C or −20°C. The CV was calculated over time for each metabolite in each patient sample.

The coefficient of variation (CV = standard deviation/mean) for each metabolite stored at either temperature was assessed (Fig. 7). The metabolites in aliquots stored at −20°C had lower CVs than those stored at 4°C, as demonstrated by the distribution of points in the violin plot in Fig. 7, where the CVs of the aliquots stored at −20°C largely fall under 0.3 while the CVs of the aliquots stored at 4°C are more widely distributed. The distribution of CVs for each metabolite during storage at −20°C remained about the same, regardless of whether the samples at −20°C were stored for 28 or 56 days or whether they were thawed once or twice (data not shown).

## DISCUSSION

Metabolite profiling complements other culture-independent approaches, such as bacterial 16S rRNA gene amplicon sequencing, in characterizing bacterial communities in biological specimens. This could aid in the identification of biomarkers of disease progression that are believed to be associated with changes in the activity of host microbial communities. In CF, for example, the production of fermentation products such as lactic acid has been found to be an indicator of pulmonary exacerbation that can be measured by GC-MS (12, 25, 26). Sample storage conditions can have a significant impact on the abundances of metabolites in biological samples, and studies assessing metabolite profiles need to account for these effects. In previous work, we observed that CF sputum sample metabolite profiles determined by LC-MS were unstable after sample storage at 4°C (21).

In this study, we captured smaller, aqueous, and more volatile metabolites in CF sputum by acetonitrile-isopropyl alcohol-water extraction, followed by untargeted GC-MS. Unfortunately, some microbial fermentation products of interest, such as 2,3-butanedione and 2,3-butanediol, are extremely volatile and were not consistently detectable by this approach. Profiles of two sputum samples stored at either 4°C or −20°C for various lengths of time were assessed. We observed that storage at −20°C yielded stable metabolite profiles with variations in metabolite abundance similar to those found in technical replicates. More specifically, the range of BC dissimilarity values among samples stored at −80°C did not increase among samples stored at −20°C. In contrast, the BC distances of samples stored at 4°C increased significantly with storage time. Of note, however, some metabolites appeared to remain stable during storage at 4°C, including some with potential as biomarkers of the CF clinical disease state, such as lactic acid.

Interindividual variation is often the most significant factor driving differences in microbial or metabolite composition between biological specimens, although the relative contributions of human and microbial metabolism to metabolite profiles are often difficult to distinguish. In this study, the metabolite profiles of samples from two individuals were consistently distinct, with the unique features of each resulting in clear demarcation by PCoA. Sample storage temperature also had a significant impact on metabolite profiles that was also apparent in PCoA. The impact of sample storage time and temperature was apparent in an analysis of total metabolite intensity versus time (Fig. S1); the total abundance of the metabolites being measured increased at 4°C but not at −20°C. Overall, these results suggest either active microbial metabolism or metabolite degradation in cells at 4°C, leading to increases in the intensity of compounds detectable by GC, including amino acids and other cellular debris.

It is also possible that changes in bacterial community structure under different storage conditions account for differences in metabolite abundance. Previous work has shown, however, that differences in community structure between sputum sample aliquots stored at room temperature and aliquots stored at other temperatures were less than the differences observed between intra- and intersample controls (27). In samples with communities not yet dominated by *P. aeruginosa*, a reduction in diversity after storage at room temperature was observed (28).

We note that although this study involved GC-MS analysis of nearly 200 samples, it was limited to two “parent” sputum samples from which dozens of aliquots were obtained, variably stored, and analyzed. While we have no reason to believe that these samples were atypical in any regard, previous work has shown that persons with CF harbor distinct microbial communities, at least during the early and intermediate stages of lung disease (6, 7). An analysis of metabolite stability (under various storage conditions) in sputum samples from a greater number of individuals is required to better demonstrate the generalizability of our findings.

In summary, storage of CF sputum samples at 4°C leads to changes in metabolite profiles within a day, with greater variation in metabolite abundances and an increase in the abundance of many of the metabolites, including several amino acids detected by untargeted GC-MS profiling. Nevertheless, several metabolites of clinical interest remain stable, including lactic acid, putrescine, and 5-aminovaleric acid. Our results suggest that CF sputum samples stored at −20°C retain stable GC-MS profiles for as long as 2 months. Freezing and thawing samples once or twice does not have a significant effect on metabolite intensities. These findings need to be considered in designing studies to assess the metabolome of microbial communities in CF airways and other environments.

## MATERIALS AND METHODS

### Sputum collection, storage, and sequencing.

CF sputum samples were collected spontaneously from two patients during the course of routine medical care. Sample collection was approved by the University of Michigan Institutional Review Board. Samples were kept on ice for up to 30 min prior to processing. An equal volume of cold sterile water was added to each sample before mechanical homogenization with a sterile tissue homogenizer (Omni International) on ice for 10 s. Each sample was divided into 100-μl aliquots, and duplicate or quadruplicate aliquots were stored at different temperatures for various lengths of time before being stored at −80°C (Fig. 2). At each time point, two aliquots stored at −20°C were thawed on ice for 30 min before being stored at −80°C.

The bacterial communities in both of the sputum samples stored continuously at −80°C were characterized as described previously (21). In brief, the V4 region of the 16S rRNA gene was amplified, sequenced, and analyzed with mothur (21).

### Metabolite extraction and metabolomic profiling.

A total of 156 sputum samples were shipped frozen on dry ice to the West Coast Metabolomics Center at the University of California (UC) Davis for untargeted GC-time of flight profiling. Metabolites were extracted from 20 μl of sputum with 1 ml of 3:3:2 acetonitrile-isopropyl alcohol-water before derivatization and GC-MS analysis by Fiehn Lab standard operating procedures (29–31). Metabolites were identified by comparison to the BinBase database. Metabolite intensities were normalized to the total intensity of the metabolites identified in each sample. Intensities were reported for 239 metabolites, of which 104 could be identified.

### Statistical analysis.

PCoA based on BC distances was used to visualize differences between sample metabolite profiles due to processing conditions. All analyses were done with R (3.2.5). PCoA was performed by using the ape library. BC distance was calculated by using the vegdist function in the vegan library. PERMANOVA was performed by using the Adonis test in the vegan package in R (32). Random-forest analysis was carried out both with the randomForest package in R by using default parameters (ntree = 500) (33) and with the rfPermute package (34), from which the figures were generated. Plots were made by using the ggplot2 and ggpubr libraries.

### Data availability.

GC-MS data have been deposited at http://datadryad.org/resource/doi:10.5061/dryad.qh100.
