# Supplementary figures and images for: Making It Last: Storage Time and Temperature Have Differential Impacts on Metabolite Profiles of Airway Samples from Cystic Fibrosis Patients
Source: mSystems. 2017 Nov 28;2(6):e00100-17. doi: 10.1128/mSystems.00100-17 (PMC5705791; doi:10.1128/mSystems.00100-17)

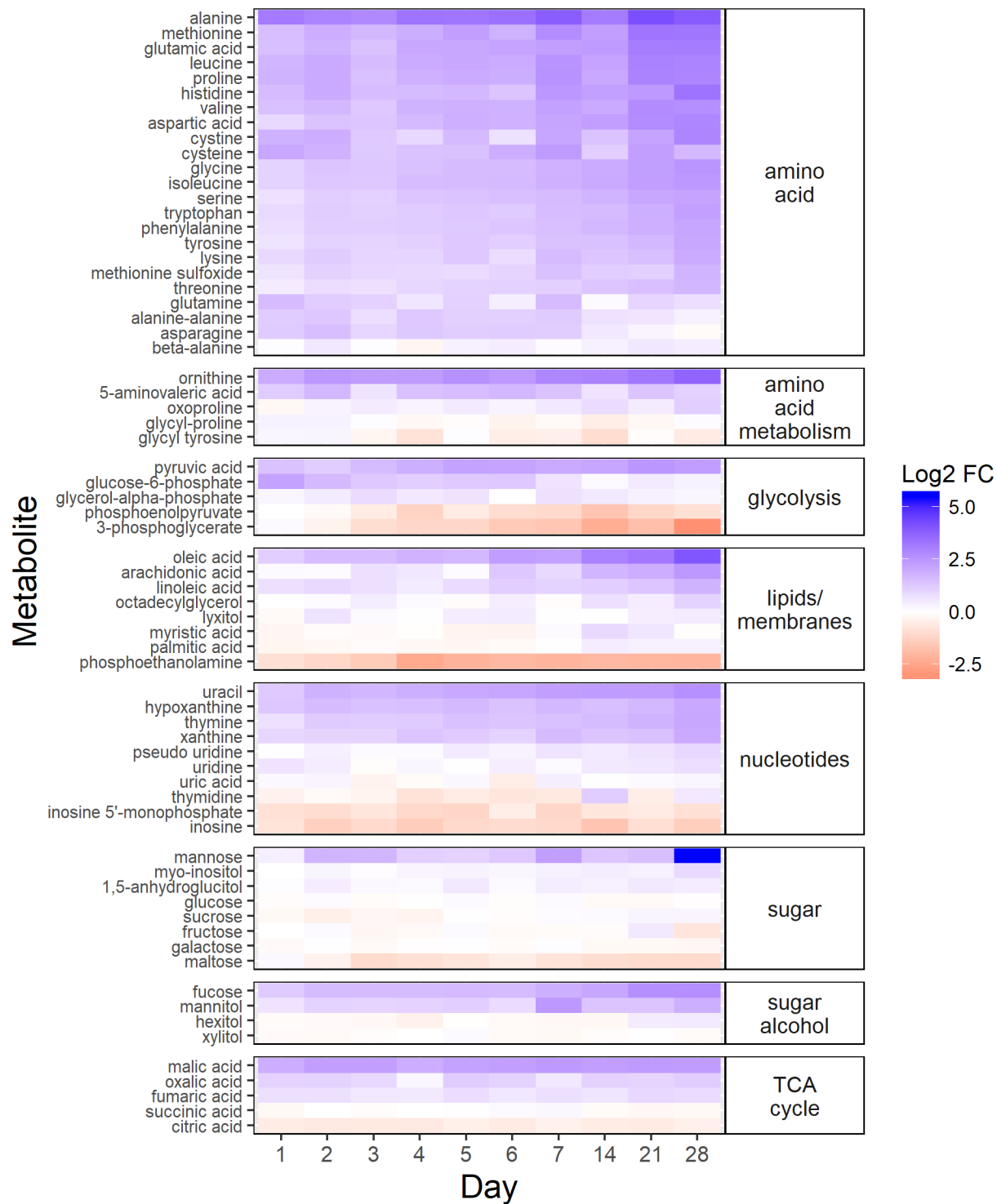

Supplement: FIG S1 [file sys006172155sf2.pdf]

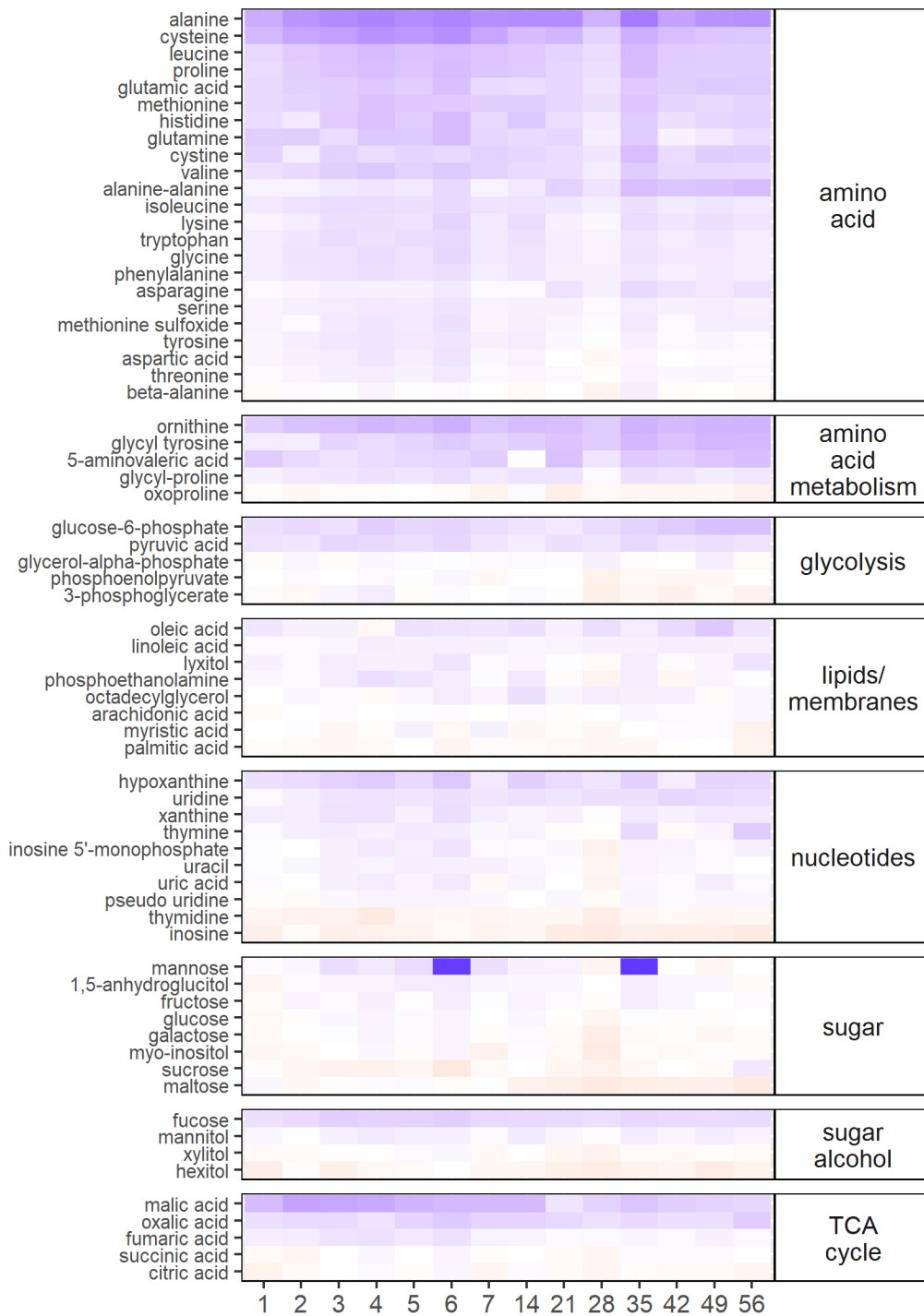

Log2 FC

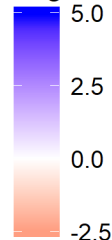

Day

Supplement: FIG S2 [file sys006172155sf3.pdf]

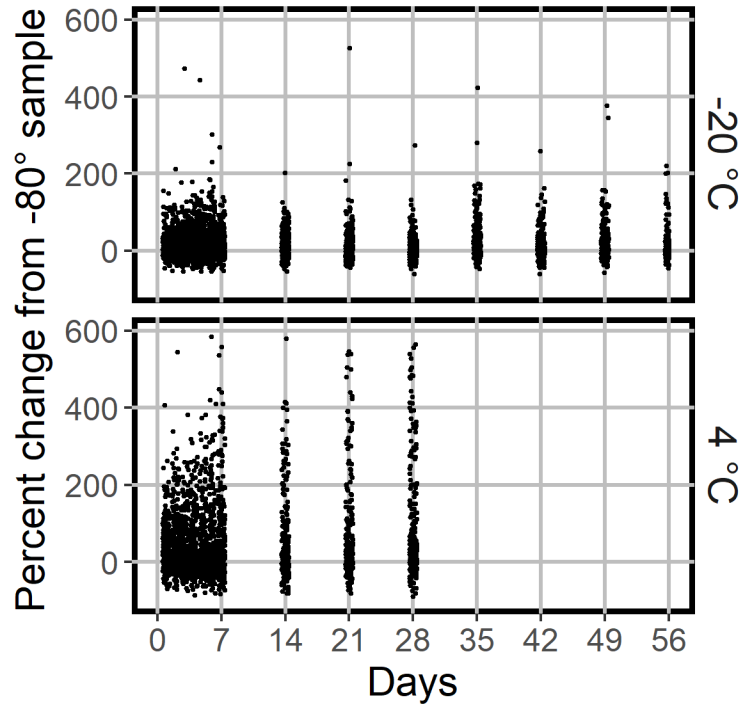

Supplement: FIG S3 [file sys006172155sf4.pdf]

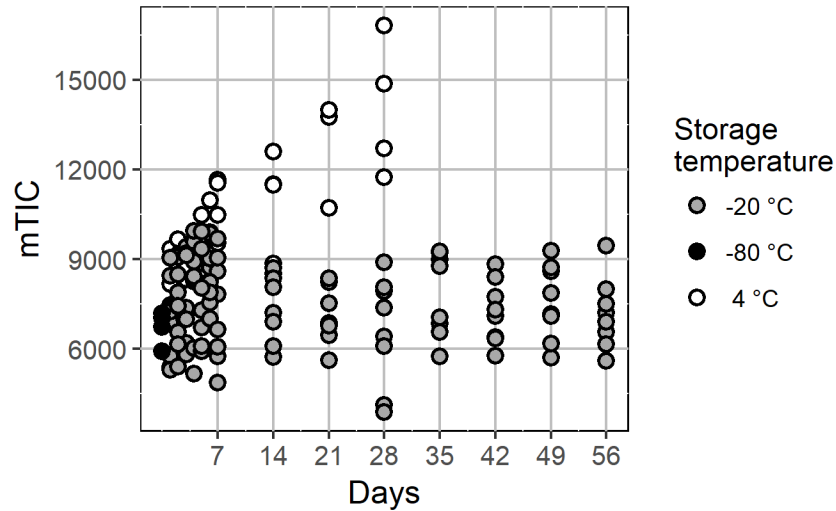

Supplement: FIG S4 [file sys006172155sf5.pdf]
