# Supplementary material for: Making It Last: Storage Time and Temperature Have Differential Impacts on Metabolite Profiles of Airway Samples from Cystic Fibrosis Patients
Source: mSystems. 2017 Nov 28;2(6):e00100-17. doi: 10.1128/mSystems.00100-17 (PMC5705791; doi:10.1128/mSystems.00100-17)
Supplement: TABLE S1 [file sys006172155st1.docx]

**Supplemental TABLE 1**

(A)

| Patient 1 | Df | Pseudo F | R2 | P-value |
| --- | --- | --- | --- | --- |
| Storage temperature | 1 | 72.3 | 0.49 | < 0.001 |
| Residuals | 74 |  | 0.51 |  |

| Patient 2 | Df | Pseudo F | R2 | P-value |
| --- | --- | --- | --- | --- |
| Storage temperature | 1 | 34.8 | 0.32 | < 0.001 |
| Residuals | 74 |  | 0.68 |  |

(B)

|  | Df | Pseudo F | R2 | P-value |
| --- | --- | --- | --- | --- |
| Storage temperature | 1 | 0.37 | 0.005 | 0.72 |
| Residuals | 70 |  | 0.995 |  |
